# Dipyridodiazepinone derivatives; synthesis and anti HIV-1 activity

**DOI:** 10.3762/bjoc.5.36

**Published:** 2009-07-22

**Authors:** Nisachon Khunnawutmanotham, Nitirat Chimnoi, Arunee Thitithanyanont, Patchreenart Saparpakorn, Kiattawee Choowongkomon, Pornpan Pungpo, Supa Hannongbua, Supanna Techasakul

**Affiliations:** 1 Chulabhorn Research Institute, Vibhavadee-Rangsit Highway, Bangkok 10210, Thailand; 2 Department of Microbiology, Faculty of Science, Mahidol University, Bangkok 10400, Thailand; 3 Department of Chemistry, Faculty of Science, Kasetsart University, Bangkok 10903, Thailand; 4 Department of Biochemistry, Faculty of Science, Kasetsart University, Bangkok 10903, Thailand; 5 Department of Chemistry, Faculty of Science, Ubonratchathani University, Ubonratchathani 34190, Thailand

**Keywords:** AIDS, anti HIV-1 RT, dipyridodiazepinone, nevirapine, synthesis

## Abstract

Ten dipyridodiazepinone derivatives were synthesized and evaluated for their anti HIV-1 reverse transcriptase activity against wild-type and mutant type enzymes, K103N and Y181C. Two of them were found to be promising inhibitors for HIV-1 RT.

## Introduction

**Figure 1:**
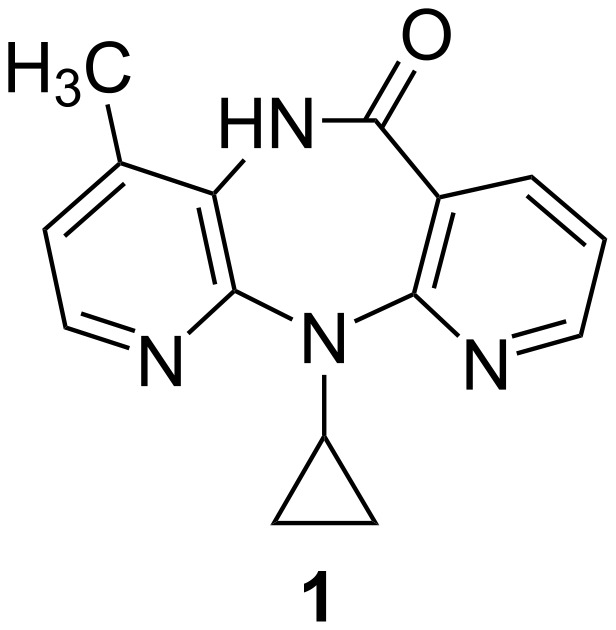
Structure of nevirapine (**1**).

**Figure 2:**
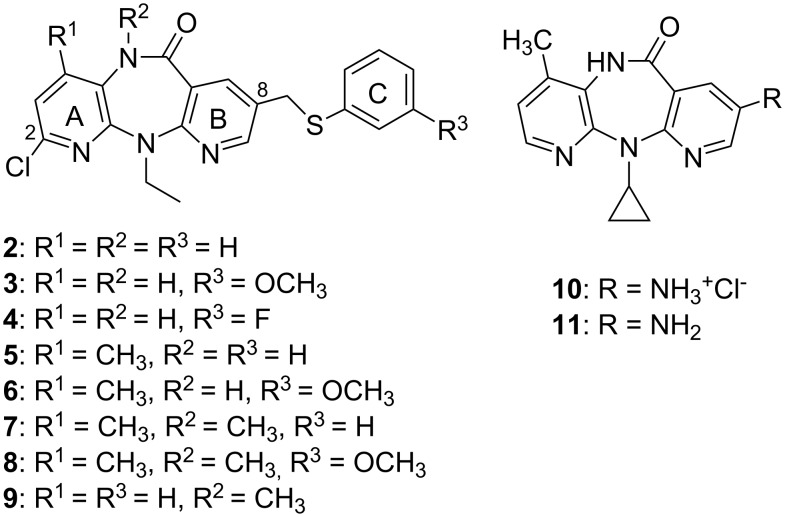
Structures of dipyridodiazepinone derivatives with promising anti-HIV activity.

Dipyridodiazepinone nevirapine (**1**) [1] (Figure 1) is a potent non-nucleoside inhibitor of human immunodeficiency virus type 1 reverse transcriptase (HIV-1 RT) and is approved as a therapeutic agent for the treatment of AIDS. In the clinic, nevirapine monotherapy results in relatively rapid drug resistance due to mutation of the RT enzyme. To develop a second-generation inhibitor with improved activity against the mutant RT enzyme, many efforts have been focused on the synthesis of dipyridodiazepinone derivatives [2–8]. On the basis of molecular modeling analysis on the wild-type (WT) and Y181C HIV-1 RT, it was found that the dipyridodiazepinone derivatives containing an unsubstituted lactam nitrogen and a 2-chloro-8-arylthiomethyl moiety, when compared with **9** [4] (Figure 2) as reference, are effective inhibitors of this mutant enzyme. Some dipyridodiazepinone derivatives containing an *N*-methylated of lactam also exhibited good potency against the WT enzyme. The 8-amino derivative of nevirapine and its hydrochloride salt also provided interesting potency. The first two compounds, **2** and **3**, were synthesized and their virustatic and virucidal activities against HIV-1 subtype E were reported previously [9]. As part of our continuing efforts directed towards the development of potential HIV-1 RT inhibitors, we have extended the synthesis of dipyridodiazepinone derivatives (Figure 2) and evaluation of their activity against wild-type RT and K103N and Y181C mutant RT enzymes.

## Results and Discussion

### Synthesis of compounds **2**–**9**

**Scheme 1:**
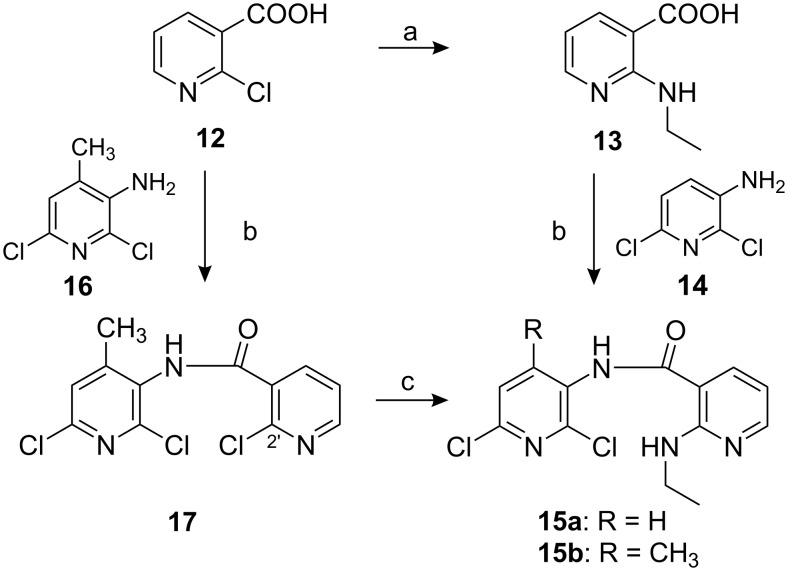
Reagents and conditions: (a) EtNH_2_, 120 °C, 4 h, 99% (b) i) (COCl)_2_, benzene, DMF, rt, 1 h; ii) amine **14** or **16**, dioxane, cyclohexane, pyridine, rt, 16 h, 80% (c) EtNH_2_, dioxane, 100 °C, 20 min, 47%.

**Scheme 2:**
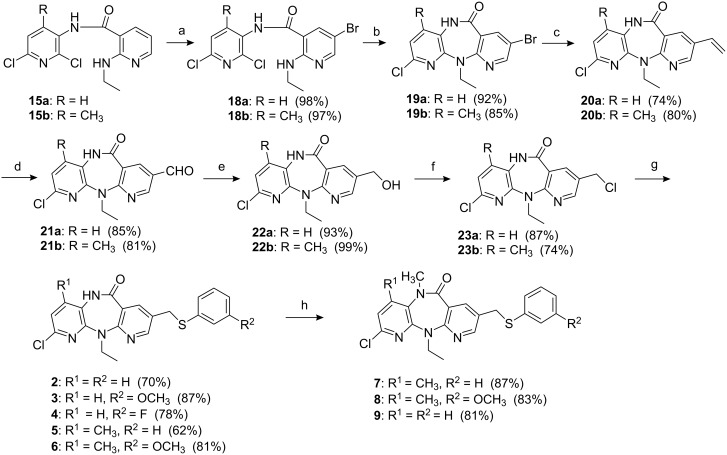
Reagents and conditions: (a) Br_2_, HOAc, KOAc, rt, 1 h; (b) NaHMDS, pyridine, 90 °C, 1 h; (c) CH_2_=CH–SnBu_3_, Pd(PPh_3_)_4_, DMF, 90 °C, 1 h; (d) O_3_, CH_2_Cl_2_/MeOH, −78 °C then PPh_3_, rt, 1 h; (e) NaBH_4_, THF, H_2_O, rt, 0.5 h; (f) SOCl_2_, CH_2_Cl_2_, Et_3_N, rt; (g) NaH, ArSH, DMF, rt, 1 h; (h) NaH, DMF, 50 °C, 0.5 h then MeI, rt, 0.5 h.

Compounds **2**–**9** were synthesized *via* efficient routes as shown in Scheme 1 and Scheme 2. The aminopyridinecarboxamide **15a** (R = H) was prepared from 2-(ethylamino)-3-pyridinecarboxylic acid (**13**) and 3-amino-2,6-dichloropyridine (**14**) (Scheme 1) [9]. However, by using the same procedure to prepare aminopyridinecarboxamide **15b** (R = CH_3_), only poor yields of **15b** were obtained. Therefore, **15b** (R = CH_3_) was prepared by formation of pyridinecarboxamide **17**, obtained from **12** and **16** [10–11], followed by the displacement of 2′ chloro by the ethylamino group.

Afterwards, the aminopyridinecarboxamide intermediates **15** were treated as previously reported [4] to give 8-arylthiomethyldipyridodiazepinones, as shown in Scheme 2.

Aminopyridinecarboxamides **15** were regioselectively brominated to produce bromo compounds **18**. The diazepinone ring was formed by treatment with sodium hexamethyldisilazane in pyridine to yield tricyclic compounds **19**. Coupling of **19** with vinyltributyltin in the presence of tetrakis(triphenylphosphine) palladium(0) provided 8-vinyl compounds **20** which underwent ozonolysis to produce aldehydes **21** in good yields. The reduction of **21** with NaBH_4_ produced alcohols **22**, which were converted to the corresponding chlorides **23** through treatment with thionyl chloride in dichloromethane. The reaction of **23a** with thiophenolate, 3-methoxythiophenolate, and 3-fluorothiophenolate in *N*,*N*-dimethylformamide yielded **2**, **3**, and **4**, respectively, whilst the reaction of **23b** with thiophenolate and 3-methoxythiophenolate yielded **5** and **6**, respectively. Methylation of the lactam nitrogen of **5** and **6** with methyl iodide provided **7** and **8**. Compound **9** was also prepared *via* methylation of **2**, which was used as the reference compound.

### Synthesis of compounds **10** and **11**

**Scheme 3:**
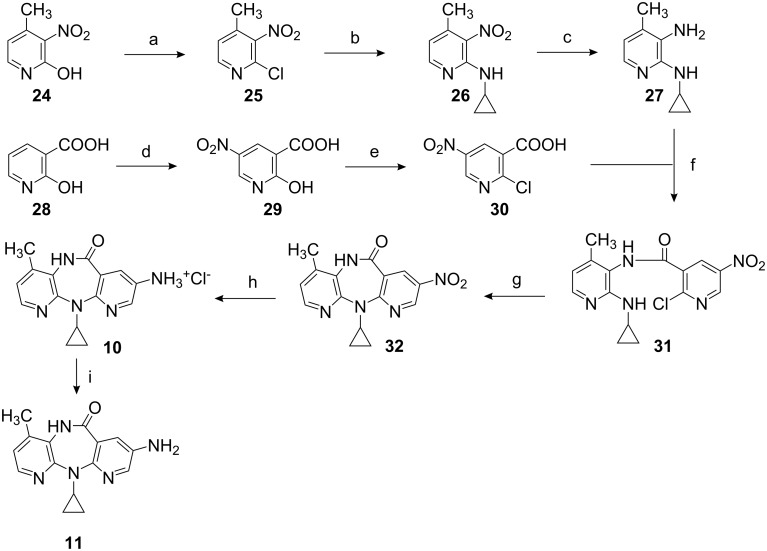
Reagents and conditions: (a) POCl_3_, 150 °C, 6 h, 85%; (b) cyclopropylamine, xylene, 105 °C, 4 h, 99%; (c) SnCl_2_·2H_2_O, conc. HCl, CH_3_COOH, rt, 3 h, 83%; (d) 69% HNO_3_, conc. H_2_SO_4_, 50 °C, 7 h, 79%; (e) POCl_3_, reflux, 4 h, 78%; (f) i) **30**, (COCl)_2_, benzene, DMF, rt, 1 h; ii) **27**, THF, DIPEA, rt, 5 h, 53%; (g) HMDS, 110 °C, 24 h, 90%; (h) SnCl_2_·2H_2_O, conc. HCl, CH_3_COOH, rt, 3h, 73%; (i) 50% aq. NaOH, rt, 1 h, 90%.

Compounds **10** and **11** were synthesized as shown in Scheme 3. The starting 3-amino-2-cyclopropylamino-4-methylpyridine (**27**) was prepared from commercially available 2-hydroxy-4-methyl-3-nitropyridine (**24**) through a sequence involving treatment with POCl_3_, followed by chloro displacement from the resulting 2-chloro compound with the aminocyclopropyl group, and finally reducing the nitro to the amino group. 2-Chloro-5-nitronicotinic acid (**30**) was prepared by nitration of commercially available 2-hydroxynicotinic acid (**28**) followed by treatment with POCl_3_. Then amine **27** and the acid **30** underwent coupling to produce carboxamide **31**. Diazepinone ring closure was performed by heating **31** in hexamethyldisilazane. Afterwards, the nitro group was reduced to produce the hydrochloride salt **10**. Treatment of **10** with 50% aqueous NaOH yielded its corresponding free amino compound **11**.

### Biological testing against HIV-1 reverse transcriptase

The results from the biological testing of all compounds synthesized, compared with nevirapine (**1**) and **9**, against the wild-type RT together with K103N and Y181C mutant RT are shown in Table 1. It was found that compounds **2**–**8** exhibited higher inhibitory activity against WT-RT and both mutant RTs compared to nevirapine. Interestingly, **5** and **6** were found to be about four times more potent against WT-RT than **9**, and they provided comparable activity against K103N mutant RT. Incorporation of a methyl group at the R^1^ position and the free N of the amide seems to be responsible for this higher activity. However, **9** showed better potency against the Y181C mutant RT compared to the other two compounds. Compound **5**, without methoxy substituent, was found to be slightly more potent than **6** except for Y181C mutant RT. Additional *N*-methyl groups in **7** and **8** led to diminished activity relative to that of **5** and **6**. **10** and **11**, 8-amino analogues of nevirapine, were found to be ineffective inhibitors.

**Table 1:** Inhibitory activity of the synthesized compounds against HIV-1 reverse transcriptase.

Compounds	IC_50_^a^ (μM)		
	
	WT	K103N	Y181C

nevirapine (**1**)	1.070 ± 0.60^b^	27.10 ± 5.20	228.5 ± 24.84
**2**	0.427 ± 0.31	6.23 ± 2.48	1.50 ± 0.34
**3**	0.757 ± 0.15	19.40 ± 2.80	2.90 ± 0.19
**4**	0.183 ± 0.10	13.90 ± 1.23	0.459 ± 0.29
**5**	0.0186 ± 0.002	0.224 ± 0.14	0.269 ± 0.08
**6**	0.0229 ± 0.01	0.428 ± 0.39	0.0593 ± 0.07
**7**	0.124 ± 0.03	4.37 ± 0.66	0.507 ± 0.36
**8**	0.0828 ± 0.03	4.59 ± 2.23	0.118 ± 0.06
**9**	0.0858 ± 0.00001	0.39 ± 0.23	0.00463 ± 0.0009
**10**	17.40 ± 2.11	62.10 ± 5.14	126.0 ± 28.03
**11**	6.05 ± 1.60	97.0 ± 16.8	61.0 ± 6.9

^a^IC_50_ is the concentration of inhibitor required for 50% inhibition of reverse transcriptase. ^b^Standard errors obtained from duplicate experiments.

### Molecular docking

**Figure 3:**
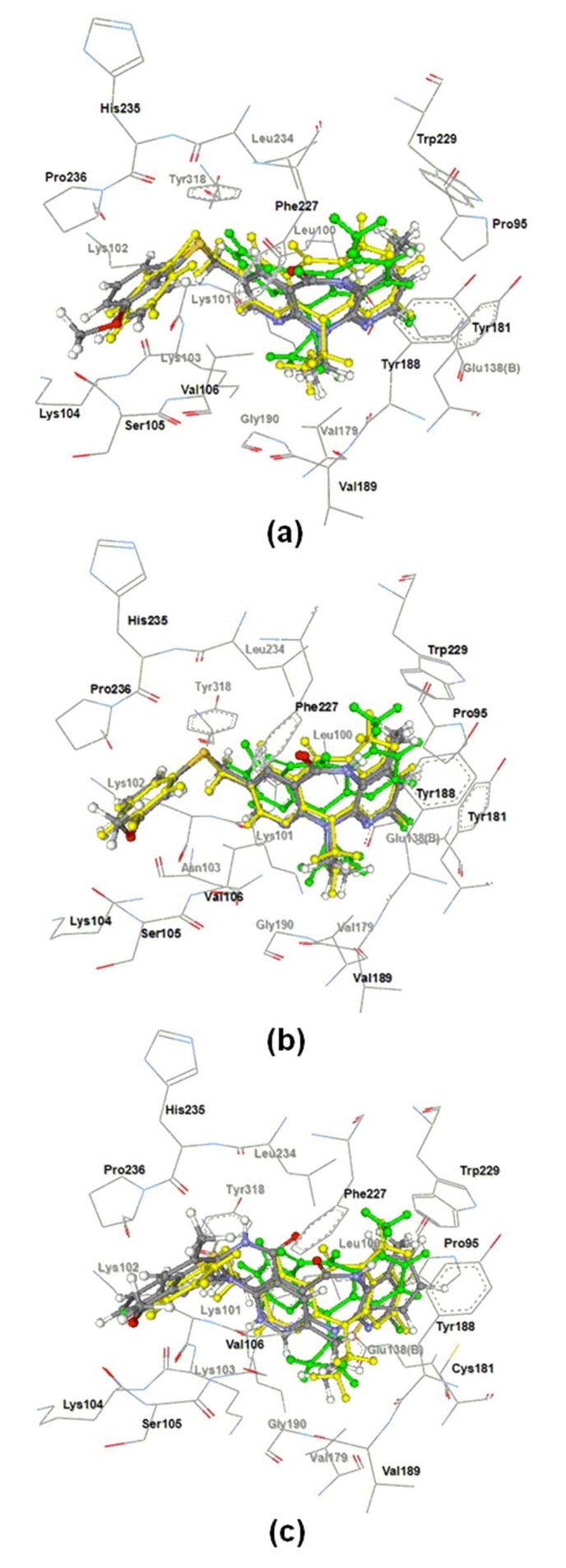
Docked orientations of nevirapine (green), **9** (yellow), **5**, and **6** (atom type color – carbon: grey, chloride: green, hydrogen: white, nitrogen: blue, oxygen: red and sulfur: yellow) in WT (a), K103N (b), and Y181C (c) binding pockets.

To understand the binding mode of the new potent derivatives **5**, **6** and **9** were docked into the HIV-1 RT binding site by using the default parameters of the GOLD v3.2 program. The wild type HIV-1 RT structure (pdb code 1klm) was taken from the protein data bank. Additionally, two HIV-1 RT mutants, K103N and Y181C, were used and analyzed by mutating positions 103 and 181 of the wild-type structure through the use of the Sybyl 7.2 program. The docked conformations of **5**, **6**, **9** and nevirapine are shown in Figure 3, and their GoldScores are presented in Table 2. In the wild-type and K103N binding sites, the docked orientations of **5**, **6** and **9** are similar to that of nevirapine. In the Y181C binding site, except for **5**, the orientations of the others were similar to nevirapine orientation. The GoldScores of **5**, **6** and **9** were higher than those of nevirapine by 17.61–24.56, 9.52–15.00 and 15.33–21.06 in the WT, K103N, and Y181C binding pockets, respectively. In the wild-type binding pocket, the H-bond interaction with the backbone oxygen atom of Lys103 was found to contain **5**, **6** and **9** but no nevirapine was present. Compounds **5**, **6** and **9** also formed an H-bond interaction with the backbone nitrogen atom of Val106. Since there is a methyl group at the R^1^ position of **5** and **6**, their docked conformations were slightly shifted below the binding pocket as compared to the docked conformation of **9**. This shift caused the formation of a stronger H-bond interaction of **5** and **6** with Lys101, Val179, Tyr188 and Val189 compared to **9**. The methyl group at R^1^ position of **5** and **6** also formed stronger H–π interaction with Trp229. Moreover, the methoxy substituent of **6** revealed a strong attractive interaction with Lys104, but the movement of ring C in **6** caused a steric interaction with the side chain of Lys102.

**Table 2:** GoldScores of nevirapine, **5**, **6** and **9** in wild-type (WT), K103N and Y181C HIV-1 RT.

Compounds	GoldScores		
	
	WT	K103N	Y181C

nevirapine	58.13 (± 0.27)^a^	58.66 (± 0.39)	56.92 (± 0.04)
**5**	72.07 (± 1.60)	79.23 (± 1.42)	66.44 (± 0.63)
**6**	79.19 (± 0.83)	83.22 (± 0.85)	71.92 (± 1.69)
**9**	73.66 (± 2.36)	76.27 (± 1.00)	68.84 (± 1.09)

^a^In parenthesis is the standard error of the GoldScore from the triplicate of docking calculations.

In the case of the docked conformations of **5**, **6** and **9** in the K103N binding pocket, the H-bond interactions with the backbone atom of Asn103 were still detected, but their H-bond interactions with Val106 were lost. Furthermore, the docked conformation of **5** showed stronger H-bond interaction with Lys101 compared to **6** and **9**. The adjustment of the ethyl group also formed an important H-bond interaction with the oxygen atom of carbonyl Val179 at distances of 2.49, 2.74 and 2.68 Å for **5**, **6** and **9** respectively. The methyl group at the R^1^ position of **5** and **6** presented the H–π interaction with side chain Trp229 closer than the hydrogen atom of **9**.

In the Y181C binding pocket, it was found that the docked conformation of **5** had a different orientation compared to **6** and **9**. Due to this orientation change of **5**, some attractive interactions found in the wild-type binding pocket were lost. It was observed that the docked conformations of **6** and **9** were aligned in the same orientation as nevirapine. For **9**, stronger attractive interactions with the backbone oxygen atoms of Val179 and Tyr188 in the Y181C binding pocket were formed as compared with the WT binding pocket. For **6**, H-bond interaction between a hydrogen atom of the methoxy group of **6** and an oxygen atom of backbone Lys104 revealed a longer bond length (3.58 Å) in the Y181C binding pocket as compared to the WT binding pocket (2.14 Å).

## Conclusion

The remarkable anti HIV-1 activity of dipyridodiazepinone derivatives, particularly compounds **5** and **6**, was presented in this study. A preliminary SAR study showed that the methyl group at the R^1^ position and the free N of amide are crucial for potent activity. This is possibly because of the strong interaction with the amino acid residue in the RT enzyme. The secondary test of these two compounds was regarded to be valuable for future investigation.

## Supporting Information

Supporting information provides details about the chemical methods, analytical data and biological testing.

File 1 Experimental part.
