# Supplementary material for: Dipyridodiazepinone derivatives; synthesis and anti HIV-1 activity
Source: Beilstein J Org Chem. 2009 Jul 22;5:36. doi: 10.3762/bjoc.5.36 (PMC2748688; doi:10.3762/bjoc.5.36)
Supplement: File 1 — Experimental part. [file Beilstein_J_Org_Chem-05-36-s001.doc]

Supporting Information

Experimental part of:

Dipyridodiazepinone derivatives; synthesis and anti HIV-1 activity

Nisachon Khunnawutmanotham^1^, Nitirat Chimnoi^1^, Arunee Thitithanyanont ^2^, Patchreenart Saparpakorn^3^, Kiattawee Choowongkomon^4^, Pornpan Pungpo^5^, Supa Hannongbua^3^ and Supanna Techasakul *^,1,3^

Address: ^1^Chulabhorn Research Institute, Vibhavadee-Rangsit Highway, Bangkok 10210, Thailand; ^2^Department of Microbiology, Faculty of Science, Mahidol University, Bangkok 10400, Thailand; ^3^Department of Chemistry, Faculty of Science, Kasetsart University, Bangkok 10903, Thailand; ^4^Department of Biochemistry, Faculty of Science, Kasetsart University, Bangkok 10903, Thailand and ^5^Department of Chemistry, Faculty of Science, Ubonratchathani University, Ubonratchathani 34190, Thailand

Email: Supanna Techasakul^*^ - supanna@cri.or.th

* Corresponding author

Chemical methods

The ^1^H (200 MHz) and ^13^C NMR (50 MHz) spectra were recorded on a Varian Gemini 2000 spectrometer referencing to solvent with the chemical shift recorded as δ values in ppm. Coupling constants (*J*) are given in Hertz, and multiplicity is defined as follows: s = singlet, d = doublet, dd = doublet of doublet, t = triplet, q = quartet, m = multiplet, br = broad. The infrared (IR) spectra were recorded in terms of cm^−1^ on a Perkin Elmer 1760x FT-IR spectrometer. Mass spectra were obtained on a Finnigan Polaris GCQ mass spectrometer, while accurate masses (HRMS) were obtained using a Bruker Micro TOF in ESI positive mode. The melting points (mp) were determined on a SMP3 melting point apparatus. Column chromatography was performed on a Scharlau silica gel 60 (70-230 mesh).

**3-Amino-2,6-dichloropyridine (14).** A solution of SnCl_2_·2H_2_O (970 mg, 4.3 mmol) in concentrated HCl (0.8 mL) was added dropwise to a stirred solution of 2,6-dichloro-3-nitropyridine (250 mg, 1.3 mmol) in acetic acid (2.6 mL), and the mixture was stirred for 3 h at room temperature. This was followed by cooling in ice bath, and subsequent addition of water. After stirring for an additional 30 min, the mixture was basified with aqueous 50% sodium hydroxide (pH 12). The reaction mixture was extracted with CH_2_Cl_2_, and the combined organic layers were washed with water, dried over Na_2_SO_4_, and concentrated under reduced pressure to produce **14** (200 mg, 94.8%) as a white solid which would be used in the next step. Recrystallization from hexane/CH_2_Cl_2_ produced needles; mp 121–122 °C. FT-IR (KBr), ν_max_: 3460, 3338, 1619, 1559, 1454, 1311, 1145, 720 cm^−1^. ^1^H NMR (CDCl_3_) *δ*: 7.11–7.01 (m, 2H), 4.16 (br s, 2H). ^13^C NMR (CDCl_3_) *δ*: 138.8, 137.3, 134.6, 125.1, 123.6 ppm. MS (EI), *m/z* (relative intensity): 162 (M^+^, 100), 126 (20), 99 (10), 64 (30). HRMS (ESI-TOF) calcd. for C_5_H_5_Cl_2_N_2_ [M+H]^+^ 162.9824; found: 162.9824.

**2-(Ethylamino)-3-pyridine carboxylic acid (13).** A stirred mixture of 2-chloronicotinic acid (**12**) (300 mg, 1.9 mmol), ethylamine (1.3 mL, 19.1 mmol) was heated at 120 °C in a sealed vessel for 4 h. After cooling down and removal of the ethylamine, the residue was purified by silica gel column chromatography (30% methanol/ethyl acetate) to produce **13** (315 mg, 99.7%) as a white solid; mp 178–180 °C. FTIR (KBr), ν_max_: 3474, 3228, 1636, 1557, 1341, 1239 cm^−1^. ^1^H NMR (DMSO-*d*_6_) *δ*: 8.20 (dd, *J* = 4.8, 1.8 Hz, 1H), 8.04 (dd, *J* = 7.7, 1.8 Hz, 1H), 6.50 (dd, *J* = 7.6, 5.1 Hz, 1H), 4.81 (br s, 1H), 3.43 (q, *J* = 6.9 Hz, 2H), 1.15 (t, *J* = 6.9 Hz, 3H). ^13^C NMR (DMSO-*d*_6_) *δ*: 169.3, 158.4, 153.0, 140.1, 110.8, 106.9, 35.1, 15.0 ppm. MS (EI), *m/z* (relative intensity): 167 (M^+^+1, 20), 151 (60), 133 (100), 122 (30), 93 (50), 78 (96). HRMS (ESI-TOF) calcd. for C_8_H_11_N_2_O_2_ [M+H]^+^ 167.0815; found: 167.0812.

**N-(2,6-Dichloro-3-pyridinyl)-2-ethylamino-3-pyridinecarboxamide (15a).** A stirred solution of **13** (315 mg, 1.9 mmol) in benzene (10 mL) was treated with oxalyl chloride (0.35 mL, 4.1 mmol), followed by a catalytic amount of *N*,*N*-dimethylformamide (2 drops). This mixture was stirred at room temperature for 1 h. Then the solvent was removed under reduced pressure to produce acid chloride as a yellow solid. This acid chloride was redissolved in 1,4-dioxane (10 mL) and added dropwise to a solution of **14** (200 mg, 1.2 mmol) in 1,4-dioxane (3 mL), cyclohexane (2 mL) and pyridine (0.2 mL, 2.4 mmol). After stirring at room temperature for 16 h, the resultant precipitate was filtered. The solid was redissolved in CH_2_Cl_2_ and washed with saturated aqueous NaHCO_3_. The organic layer was washed with water, dried over Na_2_SO_4_, and concentrated under reduced pressure. The residue was purified by silica gel column chromatography (15% EtOAc/hexane) to yield **15a** (308 mg, 80.7%) as a pale yellow solid; mp 119–120 °C. FTIR (KBr), ν_max_: 3431, 3351, 1660, 1572, 1504, 1301, 1235, 1122, 770 cm^−1^. ^1^H NMR (CDCl_3_) *δ*: 8.77 (d, *J* = 7.3 Hz, 1H), 8.33 (d, *J* = 4.4 Hz, 1H), 8.20 (s, 1H), 7.97 (br s, 1H), 7.74 (d, *J* = 7.3 Hz, 1H), 7.34 (d, *J* = 8.1 Hz, 1H), 6.60 (dd, *J* = 7.3, 4.4 Hz, 1H), 3.61–3.48 (m, 2H), 1.29 (t, *J* = 7.0 Hz, 3H). ^13^C NMR (CDCl_3_) *δ*: 166.4, 158.1, 153.4, 143.5, 138.7, 135.3, 131.2, 130.9, 123.6, 110.5, 108.0, 35.8, 14.7 ppm. MS (EI), *m/z* (relative intensity): 310 (M^+^, 20), 275 (25), 149 (35), 131 (100), 119 (20). HRMS (ESI-TOF) calcd. for C_13_H_13_Cl_2_N_4_O [M+H]^+^ 311.0461; found: 311.0464.

**N-(2,6-Dichloro-4-methyl-3-pyridinyl)-2-chloro-3-pyridinecarboxamide (17).** Using a procedure analogous to the preparation of **15a**, and starting from **12** and **16**, compound **17** was obtained in 80% as a white solid; mp 179–180 °C. FTIR (KBr), ν_max_: 3216, 1669, 1581, 1510, 1350, 1296, 1065, 856 cm^−1^. ^1^H NMR (acetone-*d*_6_) *δ*: 9.65 (br s, 1H), 8.54 (dd, *J* = 4.4, 1.5 Hz, 1H), 8.13 (dd, *J* = 7.3, 1.5 Hz, 1H), 7.56 (dd, *J* = 7.7, 4.4 Hz, 1H), 7.52 (s, 1H), 2.48 (s, 3H). ^13^C NMR (CDCl_3_) *δ*: 164.9, 153.4, 151.9, 149.3, 148.5, 148.2, 139.2, 133.3, 130.7, 126.0, 123.9, 18.8 ppm. MS (EI), *m/z* (relative intensity): 316 (M^+^, 10), 28 (100), 209 (6), 140 (35), 112 (22), 76 (29). HRMS (ESI-TOF) calcd. for C_12_H_9_N_3_OCl_3_ [M+H]^+^ 315.9806; found: 315.9813.

**N-(2,6-Dichloro-4-methyl-3-pyridinyl)-2-ethylamino-3-pyridinecarboxamide (15b).** A stirred mixture of **17** (647 mg, 2.04 mmol) and ethylamine (1.4 mL, 20.5 mmol) in 1,4-dioxane (3 mL) was heated at 100 °C in a sealed vessel for 20 min. After cooling down, water was added, and the mixture was extracted with CH_2_Cl_2_. The organic layer was washed with water, dried over Na_2_SO_4_, and concentrated under reduced pressure. The residue was purified by silica gel column chromatography (20% EtOAc/hexane) to produce **15b** (315 mg, 47%) as a white solid; mp 155–156 °C. FT-IR (KBr), ν_max_: 3394, 3214, 1625, 1582, 1499, 1332, 1255, 1109, 769 cm^−1^. ^1^H NMR (CDCl_3_) *δ*: 8.32 (dd, *J* = 4.4, 1.46 Hz, 1H), 7.97 (br s, 1H), 7.87 (dd, *J* = 7.3, 1.46 Hz, 1H), 7.60 (br s, 1H), 7.24 (s, 1H), 6.57 (dd, *J* = 7.7, 4.4 Hz, 1H), 3.58–3.45 (m, 2H), 2.34 (s, 3H), 1.26 (t, *J* = 7.0 Hz, 3H). ^13^C NMR (CDCl_3_) *δ*: 166.8, 158.1, 153.0, 150.8, 147.7, 147.0, 136.3, 129.3, 125.1, 110.4, 107.9, 36.0, 19.0, 14.7 ppm. MS (EI), *m/z* (relative intensity): 324 (M^+^, 11), 289 (28), 149 (48), 131 (100), 119 (14). HRMS (ESI-TOF) calcd. for C_14_H_15_N_4_OCl_2_ [M+H]^+^ 325.0617; found: 325.0619

**N-(2,6-Dichloro-3-pyridinyl)-5-bromo-2-ethylamino-3-pyridinecarboxamide (18a)*.*** A solution of Br_2_ (0.06 mL, 1.17 mmol) in acetic acid (1 mL) was added dropwise to a stirred solution of **15a** (355 mg, 1.14 mmol), and potassium acetate (134 mg, 1.36 mmol) in acetic acid (15 mL). After 15 min, the reaction mixture was added with water, and the precipitate was collected through suction filtration. It was then washed with water for several times to produce **18a** (435 mg, 98.0%) as a yellow solid; mp 193–194 °C. FT-IR (KBr), ν_max_: 3439, 3336, 1673, 1575, 1506, 1302, 1248, 787, 528 cm^−1^. ^1^H NMR (CDCl_3_) *δ*: 8.69 (d, *J* = 8.8 Hz, 1H), 8.33 (d, *J* = 2.2 Hz, 1H), 8.10 (br s, 1H), 7.89 (br s, 1H), 7.79 (d, *J* = 2.2 Hz, 1H), 7.30 (d, *J* = 8.1 Hz, 1H), 3.58–3.44 (m, 2H), 1.27 (t, *J* = 7.3 Hz, 3H). ^13^C NMR (CDCl_3_) *δ*: 165.4, 156.5, 154.2, 139.1, 137.4, 131.7, 130.7, 123.8, 109.6, 103.9, 36.1, 14.6 ppm. MS (EI), *m/z* (relative intensity): 388 (M^+^, 60), 373 (15), 353 (20), 227 (70), 209 (60). HRMS (ESI-TOF) calcd. for C_13_H_12_BrCl_2_N_4_O [M+H]^+^ 388.9566; found: 388.9579.

**N-(2,6-Dichloro-4-methyl-3-pyridinyl)-5-bromo-2-ethylamino-3-pyridinecarboxamide (18b).** Using a procedure analogous to the preparation of **18a**, and starting from **15b**, **18b** was obtained in 97.5% as a yellow solid; mp 186–187 °C. FT-IR (KBr), ν_max_: 3417, 3259, 1635, 1583, 1497, 1350, 1246, 788, 521 cm^−1^. ^1^H NMR (CDCl_3_) *δ*: 8.32 (d, *J* = 2.2 Hz, 1H), 7.93 (d, *J* = 2.2 Hz, 1H and br s, 1H), 7.53 (br s, 1H), 7.25 (s, 1H), 3.55–3.41 (m, 2H), 2.33 (s, 3H), 1.24 (t, *J* = 7.3 Hz, 3H). ^13^C NMR (CDCl_3_) *δ*: 165.7, 156.1, 152.8, 150.8, 148.0, 147.1, 138.6, 128.8, 125.2, 109.4, 103.6, 36.3, 19.0, 14.5 ppm. MS (EI), *m/z* (relative intensity): 402 (M^+^, 20), 367 (27), 323 (8), 227 (77), 209 (80). HRMS (ESI-TOF) calcd. for C_14_H_14_N_4_OCl_2_Br [M+H]^+^ 402.9723; found: 402.9730.

**8-Bromo-2-chloro-5,11-dihydro-11-ethyl-6*H*-dipyrido[3,2-*b*:2’,3’-*e*][1,4]diazepin-6-one (19a).** A solution of **18a** (440 mg, 1.13 mmol) in pyridine (10 mL) was heated at 90 °C under nitrogen atmosphere. Then the solution was added sodium bis(trimethylsilyl)amide (0.6 M in toluene, 6.3 mL, 3.7 mmol), and the mixture was stirred at 90 °C for 15 min. After cooling down, the mixture was poured into ice water and stirred for 2 h. The precipitate was filtered and washed with water to produce **19a** (370 mg, 92.8%) as a yellow solid; mp 263–264 °C. FT-IR (KBr), ν_max_: 3195, 3074, 2972, 1665, 1575, 1456, 1381, 1226, 687, 630 cm^−1^. ^1^H NMR (CDCl_3_) *δ*: 8.53 (br s, 1H), 8.49 (d, *J* = 2.2 Hz, 1H), 8.24 (d, *J* = 2.2 Hz, 1H), 7.27 (d, *J* = 8.1 Hz, 1H), 7.06 (d, J = 8.1 Hz, 1H), 4.81 (q, *J* = 7.3 Hz, 2H), 1.25 (t, *J* = 7.3 Hz, 3H). ^13^C NMR (DMSO-*d*_6_) *δ*: 165.4, 156.8, 151.8, 150.5, 143.0, 142.6, 133.3, 126.3, 122.2, 120.4, 113.6, 41.5, 13.4 ppm. MS (EI), *m/z* (relative intensity): 352 (M^+^, 80), 337 (42), 324 (75), 309 (20). HRMS (ESI-TOF) calcd. for C_13_H_11_BrClN_4_O [M+H]^+^ 352.9799; found: 352.9803.

**8-Bromo-2-chloro-5,11-dihydro-11-ethyl-4-methyl-6*H*-dipyrido[3,2-*b*:2’,3’-*e*][1,4]diazepin-6-one (19b).** Using a procedure analogous to the preparation of **19a**, and starting from **18b**, after purification with silica gel column chromatography (20% EtOAc/hexane), **19b** was obtained in 85% as a yellow solid; mp 222–223 °C. FT-IR (KBr), ν_max_: 3181, 3065, 2965, 1664, 1578, 1440, 1376, 1229, 675, 640 cm^−1^. ^1^H NMR (CDCl_3_) *δ*: 8.74 (br s, 1H), 8.47 (d, *J* = 2.9 Hz, 1H), 8.18 (d, *J* = 2.9 Hz, 1H), 6.97 (s, 1H), 4.14 (q, *J* = 7.3 Hz, 2H), 2.39 (s, 3H), 1.23 (t, *J* = 7.3 Hz, 3H). ^13^C NMR (CDCl_3_) *δ*: 167.5, 158.0, 152.5, 152.3, 145.3, 142.5(2C), 123.8, 121.6(2C), 114.0, 42.1, 17.8, 13.6 ppm. MS (EI), *m/z* (relative intensity): 366 (M^+^, 64), 351 (45), 338 (70), 324 (25). HRMS (ESI-TOF) calcd. for C_14_H_13_N_4_OClBr [M+H]^+^ 366.9956; found: 366.9960.

**2-Chloro-5,11-dihydro-11-ethyl-8-vinyl-6*H*-dipyrido[3,2-*b*:2’,3’-*e*][1,4]diazepin-6-one (20a).** A solution of **19a** (500 mg, 1.4 mmol) in *N*,*N*-dimethylformamide (7 mL) was treated with tetrakis(triphenylphosphine)­palladium(0) (70 mg, 0.06 mmol) followed by vinyl tributyltin (1 mL, 3.42 mmol), and heated at 100 °C under N_2_ atmosphere for 0.5 h. After cooling down, the reaction mixture was poured into water and extracted with ethyl acetate. The combined organic layers were washed with 15% aqueous ammonia, brine, and water, and then dried over Na_2_SO_4_ and concentrated under reduced pressure. The residue was purified by silica gel column chromatography (30% EtOAc/hexane) to produce **20a** (318 mg, 74.6%) as a yellow solid; mp 201–202 °C. FT-IR (KBr), ν_max_: 3195, 2965, 1665, 1585, 1455, 1383, 1239, 688 cm^−1^. ^1^H NMR (CDCl_3_) *δ*: 9.37 (s, 1H), 8.46 (d, *J* = 2.2 Hz, 1H), 8.20 (d, *J* = 2.2 Hz, 1H), 7.35 (d, *J* = 8.1 Hz, 1H), 7.04 (d, *J* = 8.1 Hz, 1H), 6.66 (dd, *J* = 17.6, 11.0 Hz, 1H), 5.78 (d, *J* = 17.6 Hz, 1H), 5.35 (d, *J* = 11.0 Hz, 1H), 4.22 (q, *J* = 7.3 Hz, 2H), 1.27 (t, *J* = 7.3 Hz, 3H). ^13^C NMR (CDCl_3_) *δ*: 168.8, 157.9, 151.9, 150.1, 145.1, 137.7, 131.9, 131.8, 128.8, 125.0, 119.7, 119.6, 115.8, 42.1, 13.7 ppm. MS (EI), *m/z* (relative intensity): 300 (M^+^, 100), 285 (35), 272 (92), 257 (35). HRMS (ESI-TOF) calcd. for C_15_H_14_ClN_4_O [M+H]^+^ 301.0851; found: 301.0855.

**2-Chloro-5,11-dihydro-11-ethyl-4-methyl-8-vinyl-6*H*-dipyrido[3,2-*b*:2’,3’-*e*][1,4]diazepin-6-one (20b).** Using a procedure analogous to the preparation of **20a**, and starting from **19b**, **20b** was obtained in 80% as a pale brown solid; mp 185–186 °C (dec.). FT-IR (KBr), ν_max_: 3189, 2968, 1656, 1594, 1459, 1376, 1232 cm^−1^. ^1^H NMR (CDCl_3_) *δ*: 8.43 (d, *J* = 2.2 Hz, 1H), 8.41 (s, 1H), 8.16 (d, *J* = 2.2 Hz, 1H), 6.94 (s, 1H), 6.65 (dd, *J* = 17.6, 11 Hz, 1H), 5.76 (d, *J* = 17.6 Hz, 1H), 5.33 (d, *J* = 11 Hz, 1H), 4.18 (q, *J* = 7.3 Hz, 2H), 2.38 (s, 3H), 1.24 (t, *J* = 7.3 Hz, 3H). ^13^C NMR (CDCl_3_) *δ*: 168.8, 158.5, 152.6, 149.9, 145.0, 142.3, 137.2, 131.9, 128.7, 123.8, 121.2, 120.0, 115.6, 42.0, 17.8, 13.6 ppm. MS (EI), *m/z* (relative intensity): 314 (M^+^, 100), 299 (43), 286 (80), 271 (34), 257 (11). HRMS (ESI-TOF) calcd. for C_16_H_16_N_4_OCl [M+H]^+^ 315.1007; found: 315.1009.

**2-Chloro-5,11-dihydro-11-ethyl-8-formyl-6*H*-dipyrido[3,2-*b*:2’,3’-*e*][1,4]diazepin-6-one (21a).** A –78 °C cooled solution of **20a** (343 mg, 1.14 mmol) in 1:1 dichloromethane-methanol (20 mL) was treated with O_3_ for 30 min. After the completion of the reaction, the solution was purged with O_2_ for 5 min. Then the reaction mixture was stirred with triphenylphosphine (598 mg, 2.28 mmol) for an additional 1 h at room temperature. The solvent was removed under reduced pressure, and the residue was purified by silica gel column chromatography (30% EtOAc/hexane) to yield **21a** (295 mg, 85.4%) as a yellow solid; mp 216–217 °C. FT-IR (KBr), ν_max_: 3203, 2939, 1702, 1674, 1596, 1456, 1354, 1231, 975, 692. ^1^H NMR (CDCl_3_) *δ*: 10.02 (s, 1H), 9.53 (s, 1H), 8.60 (d, *J* = 2.2 Hz, 1H), 8.64 (d, *J* = 2.2 Hz, 1H), 7.41 (d, *J* = 8.1 Hz, 1H), 7.13 (d, J = 8.1 Hz, 1H), 4.34 (q, *J* = 7.0 Hz, 2H), 1.32 (t, *J* = 7.0 Hz, 3H). ^13^C NMR (CDCl_3_) *δ*: 188.6, 168.1, 162.1, 153.8, 149.9, 145.3, 142.3, 132.3, 126.9, 125.0, 120.8, 118.8, 43.0, 13.7 ppm. MS (EI), *m/z* (relative intensity): 302 (M^+^, 80), 287 (30), 274 (100), 260 (30), 245 (35). HRMS (ESI-TOF) calcd. for C_14_H_12_ClN_4_O_2_ [M+H]^+^ 303.0643; found: 303.0645.

**2-Chloro-5,11-dihydro-11-ethyl-8-formyl-4-methyl-6*H*-dipyrido[3,2-*b*:2’,3’-*e*][1,4]diazepin-6-one (21b).** Using a procedure analogous to the preparation of **21a**, and starting from **20b**, compound **21b** was obtained in 81% as a yellow solid; mp 207–208 °C. FT-IR (KBr), ν_max_: 3181, 2964, 1697, 1693, 1591, 1459, 1355, 1228 , 903, 807 cm^−1^. ^1^H NMR (CDCl_3_) *δ*: 10.01 (s, 1H), 8.89 (d, *J* = 2.2 Hz, 1H), 8.83 (s, 1H), 8.57 (d, *J* = 2.2 Hz, 1H), 7.02 (s, 1H), 4.30 (q, *J* = 7.3 Hz, 2H), 2.43 (s, 3H), 1.29 (t, *J* = 7.3 Hz, 3H). ^13^C NMR (CDCl_3_) *δ*: 188.6, 167.7, 162.6, 153.7, 150.9, 145.3, 142.9, 142.0, 127.0, 123.9, 122.2, 119.5, 42.9, 17.9, 13.7 ppm. MS (EI), *m/z* (relative intensity): 316 (M^+^, 100), 301 (40), 288 (83), 273 (22), 259 (14). HRMS (ESI-TOF) calcd. for C_15_H_14_N_4_O_2_Cl [M+H]^+^ 317.0800; found: 317.0806.

**2-Chloro-5,11-dihydro-11-ethyl-8-hydroxymethyl-6*H*-dipyrido[3,2-*b*:2’,3’-*e*][1,4]diazepin-6-one (22a).** A solution of **21a** (228 mg, 0.75 mmol) in tetrahydrofuran (12 mL) was added with water (0.1 mL) and sodium borohydride (28.5 mg, 0.75 mmol). The mixture was stirred for 0.5 h, then diluted with additional water. Tetrahydrofuran was removed under reduced pressure, and the precipitate was filtered and washed with water to yield **22a** (214 mg, 93%) as a white solid; mp 197–198 °C. FT-IR (KBr), ν_max_: 3319, 3191, 2959, 1666, 1590, 1456, 1390, 1232, 1041 cm^−1^. ^1^H NMR (acetone-*d*_6_) *δ*: 9.50 (br s, 1H), 8.46 (d, *J* = 2.2 Hz, 1H), 8.11 (d, *J* = 2.2 Hz, 1H), 7.60 (d, *J* = 8.1 Hz, 1H), 7.18 (d, *J* = 8.1 Hz, 1H), 4.65 (d, *J* = 5.13 Hz, 2H), 4.56 (t, *J* = 5.86 Hz, 1H), 4.15 (q, *J* = 7.3 Hz, 2H), 1.21 (t, *J* = 7.3 Hz, 3H). ^13^C NMR (acetone-*d*_6_) *δ*: 167.8, 158.7, 152.7, 151.0, 144.5, 140.3, 134.1, 133.4, 127.3, 121.2, 120.5, 61.5, 42.4, 14.0 ppm. MS (EI), *m/z* (relative intensity): 304 (M^+^, 69), 289 (39), 276 (100), 261 (29), 247 (20), 164 (31). HRMS (ESI-TOF) calcd. for C_14_H_14_ClN_4_O_2_ [M+H]^+^ 305.0800; found: 305.0805.

**2-Chloro-5,11-dihydro-11-ethyl-8-hydroxymethyl-4-methyl-6*H*-dipyrido[3,2-*b*:2’,3’-*e*][1,4]diazepin-6-one (22b).** Using a procedure analogous to the preparation of **22a**, and starting from **21b**, compound **22b** was obtained in 99% as a white solid; mp 259–260 °C. FT-IR (KBr), ν_max_: 3352, 3191, 1651, 1594, 1477, 1386, 1228, 1044 cm^−1^. ^1^H NMR (DMSO-*d*_6_) *δ*: 10.01 (s, 1H), 8.39 (d, *J* = 2.2 Hz, 1H), 7.97 (d, *J* = 2.2 Hz, 1H), 7.19 (s, 1H), 5.33 (t, *J* = 5.86 Hz, 1H), 4.48 (d, *J* = 5.86 Hz, 2H), 3.99 (q, *J* = 7.3 Hz, 2H), 2.33 (s, 1H), 1.14 (t, *J* = 7.3 Hz, 3H). ^13^C NMR (DMSO-*d*_6_) *δ*: 166.9, 157.4, 153.0, 149.4, 144.7, 143.0, 138.7, 133.3, 124.6, 121.2, 120.7, 59.7, 41.0, 17.5, 14.0 ppm. MS (EI), *m/z* (relative intensity): 318 (M^+^, 100), 303 (45), 290 (88), 275 (20), 261 (9). HRMS (ESI-TOF) calcd. for C_15_H_16_N_4_O_2_Cl [M+H]^+^ 319.0956; found: 319.0955.

**2-Chloro-5,11-dihydro-11-ethyl-8-chloromethyl-6*H*-dipyrido[3,2-*b*:2’,3’-*e*][1,4]diazepin-6-one (23a).** A suspension of **22a** (177 mg, 0.58 mmol) in dichloromethane (100 mL) was treated with thionyl chloride (0.3 mL) followed by triethylamine (1 mL). The reaction mixture was stirred at room temperature for 1 h, and a clear solution was eventually obtained. Afterwards, the saturated aqueous NaHCO_3_ was added, and the mixture was extracted with CH_2_Cl_2_. The organic layer was washed with water, dried over Na_2_SO_4_, and concentrated under reduced pressure. The residue was purified by silica gel column chromatography (20% EtOAc/hexane) to yield **23a** (163 mg, 87%) as a pale yellow solid; mp 226–227 °C. FT-IR (KBr), ν_max_: 3195, 2969, 1671, 1588, 1455, 1382, 1247, 699 cm^−1^. ^1^H NMR (CDCl_3_) *δ*: 9.15 (br s, 1H), 8.47 (d, *J* = 2.9 Hz, 1H), 8.19 (d, *J* = 2.9 Hz, 1H), 7.34 (d, *J* = 8.1 Hz, 1H), 7.06 (d, *J* = 8.1 Hz, 1H), 4.58 (s, 2H), 4.23 (q, *J* = 7.3 Hz, 2H), 1.27 (t, *J* = 7.3 Hz, 3H). ^13^C NMR (CDCl_3_) *δ*: 168.3, 158.7, 151.7, 151.5, 145.2, 141.4, 131.9, 128.3, 125.0, 119.9, 119.6, 42.3, 42.2, 13.7 ppm. MS (EI), *m/z* (relative intensity): 322 (M^+^, 54), 307 (31), 294 (62), 287 (37), 259 (100), 244 (28), 231 (21). HRMS (ESI-TOF) calcd. for C_14_H_13_Cl_2_N_4_O [M+H]^+^ 323.0461; found: 323.0470.

**2-Chloro-5,11-dihydro-11-ethyl-8-chloromethyl-4-methyl-6*H*-dipyrido[3,2-*b*:2’,3’-*e*][1,4]diazepin-6-one (23b).** Using a procedure analogous to the preparation of **23a**, and starting from **22b**, compound **23b** was obtained in 74% as a white solid; mp 229–230 °C. FT-IR (KBr), ν_max_: 3180, 2970, 1651, 1603, 1465, 1382, 1284, 707 cm^−1^. ^1^H NMR (CDCl_3_) *δ*: 8.57 (br s, 1H), 8.45 (d, *J* = 2.2 Hz, 1H), 8.14 (d, *J* = 2.2 Hz, 1H), 6.96 (s, 1H), 4.56 (s, 2H), 4.19 (q, *J* = 7.3 Hz, 2H), 2.39 (s, 3H), 1.24 (t, *J* = 7.3 Hz, 3H). ^13^C NMR (CDCl_3_) *δ*: 168.2, 159.2, 152.4, 151.6, 145.2, 142.4, 141.0, 128.4, 123.9, 121.5, 120.1, 42.3, 42.1, 17.8, 13.7 ppm. MS (EI), *m/z* (relative intensity): 336 (M^+^, 79), 321 (56), 308 (75), 301 (38), 293 (19), 273 (100), 258 (15), 245 (18). HRMS (ESI-TOF) calcd. for C_15_H_15_N_4_OCl_2_ [M+H]^+^ 337.0617; found: 337.0624.

**2-Chloro-5,11-dihydro-11-ethyl-8-[(phenylthio)-methyl]-6*H*-dipyrido[3,2-*b*:2’,3’-*e*][1,4]diazepin-6-one (2).** A solution of thiophenol (0.1 mL, 0.97 mmol) in *N*,*N*-dimethylformamide (2 mL) was treated with sodium hydride (60% suspension in oil, 65 mg, 1.6 mmol) under N_2_ atmosphere. After 10 min, a solution of **23a** (100 mg, 0.31 mmol) in *N*,*N*-dimethylformamide (3 mL) was added, and the mixture was stirred at room temperature for 1 h. The reaction was quenched by adding water, and the mixture was extracted with ethyl acetate. The organic layer was washed with water, dried over Na_2_SO_4_, and concentrated under reduced pressure. The residue was purified by silica gel column chromatography (20% EtOAc/hexane) to give **2** (85.5 mg, 70%) as a yellow solid; mp 188.5–189.5 °C. FT-IR (KBr), ν_max_: 3182, 2965, 1665, 1585, 1455, 1383, 1239, 739, 688 cm^−1^. ^1^H NMR (CDCl_3_) *δ*: 9.33 (s, 1H), 8.29 (d, *J* = 2.2 Hz, 1H), 8.09 (d, *J* = 2.2 Hz, 1H), 7.34–7.20 (m, 6H), 7.04 (d, *J* = 8.1 Hz, 1H), 4.18 (q, *J* = 7.3 Hz, 2H), 4.05 (s, 2H), 1.23 (t, *J* = 7.3 Hz, 3H). ^13^C NMR (CDCl_3_) *δ*: 168.6, 157.8, 151.8, 145.1, 141.2, 134.9, 131.8, 130.6(2C), 129.1(2C), 128.6, 127.0(2C), 125.1, 119.7, 119.6, 42.0, 35.6, 13.6 ppm. MS (EI), *m/z* (relative intensity): 396 (M^+^, 7), 287 (100), 259 (52), 231 (14). HRMS (ESI-TOF) calcd. for C_20_H_18_ClN_4_OS [M+H]^+^ 397.0884; found: 397.0894.

**2-Chloro-5,11-dihydro-11-ethyl-8-{[(3-methoxyphenyl)thio]methyl}-6*H*-dipyrido[3,2-*b*:2’,3’-*e*][1,4]diazepin-6-one (3).** Using a procedure analogous to the preparation of **2**, and starting from **23a** and 3-methoxythiophenol, **3** was obtained in 87% as yellow crystals; mp 188–189 °C. FT-IR (KBr), ν_max_: 3181, 2965, 1662, 1589, 1458, 1385, 1229, 1044, 767, 693 cm^−1^. ^1^H NMR (CDCl_3_) *δ*: 9.56 (br s, 1H), 8.30 (d, *J* = 2.2 Hz, 1H), 8.08 (d, *J* = 2.2 Hz, 1H), 7.33 (d, *J* = 8.1 Hz, 1H), 7.18 (t, *J* = 8.1 Hz, 1H), 7.04 (d, *J* = 8.1 Hz, 1H), 6.90–6.72 (m, 3H), 4.06 (s, 2H), 3.73 (s, 3H), 1.23 (t, *J* = 7.3 Hz, 3H). ^13^C NMR (CDCl_3_) *δ*: 168.8, 159.9, 157.9, 151.8(2C), 145.1, 141.2, 136.2, 131.8, 129.9, 128.6, 125.2, 122.6, 119.7, 119.6, 115.8, 112.9, 55.3, 42.1, 35.4, 13.6 ppm. MS (EI), *m/z* (relative intensity): 426 (M^+^, 6), 287 (100), 259 (57), 231 (15). HRMS (ESI-TOF) calcd. for C_21_H_20_ClN_4_O_2_S [M+H]^+^ 427.0990; found: 427.0988.

**2-Chloro-5,11-dihydro-11-ethyl-8-{[(3-fluorophenyl)thio]methyl}-6*H*-dipyrido[3,2-*b*:2’,3’-*e*][1,4]diazepin-6-one (4).** Using a procedure analogous to the preparation of **2**, and starting from **23a** and 3-fluorothiophenol, **4** was obtained in 78% as yellow crystals; mp 179–180 °C. FT-IR (KBr), ν_max_: 3189, 2935, 1663, 1579, 1454, 1384, 1250, 1145, 776 cm^−1^. ^1^H NMR (CDCl_3_) *δ*: 9.14 (s, 1H), 8.34 (s, 1H), 8.11 (s, 1H), 7.33 (d, *J* = 7.8 Hz, 1H), 7.26–7.20 (m, 1H), 7.07–7.00 (m, 3H), 6.89 (dt, J = 8.3, 2.2 Hz, 1H), 4.20 (q, *J* = 6.9 Hz, 2H), 4.08 (s, 2H), 1.24 (t, *J* = 6.8 Hz, 3H). ^13^C NMR (CDCl_3_) *δ*: 168.5, 164 (d, ^1^*J*_F,C_ = 220), 157.9, 151.6(2C), 145.1, 141.4, 137.2, 131.9, 130.4 (d, ^3^J_F,CH_ = 8.5), 127.9, 125.3, 125.0, 119.8, 119.7, 116.5 (d, ^2^*J*_F,CH_ = 23.1), 113.8 (d, ^2^*J*_F,CH_ = 21), 42.1, 35.0, 13.6 ppm. MS (EI), *m/z* (relative intensity): 414 (M^+^, 10), 287 (100), 259 (47), 231 (16). HRMS (APCI-TOF) calcd. for C_20_H_17_N_4_OSClF [M+H]^+^ 415.0790; found: 415.0787.

**2-Chloro-5,11-dihydro-11-ethyl-4-methyl-8-[{(phenyl)thio]methyl}-6*H*-dipyrido[3,2-*b*:2’,3’-*e*][1,4]diazepin-6-one (5).** Using a procedure analogous to the preparation of **2**, and starting from **23b** and thiophenol, **5** was obtained in 62% as white crystals; mp 166–167 °C. FT-IR (KBr), ν_max_: 3174, 2965, 1651, 1593, 1461, 1378, 1231, 737, 689 cm^−1^. ^1^H NMR (CDCl_3_) *δ*: 8.60 (br s, 1H), 8.27 (d, *J* = 2.2 Hz, 1H), 8.05 (d, *J* = 2.2 Hz, 1H), 7.31–7.19 (m, 5H), 6.93 (s, 1H), 4.14 (q, *J* = 7.3 Hz, 2H), 4.05 (s, 2H), 2.37 (s, 3H), 1.21 (t, *J* = 7.3 Hz, 3H). ^13^C NMR (CDCl_3_) *δ*: 168.5, 158.3, 152.6, 151.6, 145.0, 142.3, 140.9, 135.0, 130.5(2C), 129.1(2C), 128.6, 127.0, 124.0, 121.3, 120.1, 42.0, 35.6, 17.8, 13.6 ppm. MS (EI), *m/z* (relative intensity): 410 (M^+^, 10), 301 (100), 273 (30), 245 (6). HRMS (ESI-TOF) calcd. for C_21_H_20_N_4_OSCl [M+H]^+^ 411.1041; found: 411.1039.

**2-Chloro-5,11-dihydro-11-ethyl-4-methyl-8-[{(3-methoxyphenyl)thio]­methyl}-6*H*-dipyrido[3,2-*b*:2’,3’-*e*][1,4]diazepin-6-one (6*).*** Using a procedure analogous to the preparation of **2**, and starting from **23b** and 3-methoxythiophenol, **6** was obtained in 81% as white crystals; mp 176–177 °C. FT-IR (KBr), ν_max_: 3187, 1661, 1591, 1571, 1461, 1385, 1235, 765, 684 cm^−1^. ^1^H NMR (CDCl_3_) *δ*: 8.29 (d, *J* = 2.2 Hz, 1H), 8.25 (br s, 1H), 8.05 (d, *J* = 2.2 Hz, 1H), 7.18 (dd, *J* = 8.1, 8.1 Hz, 1H), 6.94 (s, 1H), 6.90–6.71 (m, 3H), 4.14 (q, *J* = 7.3 Hz, 2H), 4.05 (s, 2H), 3.72 (s, 3H), 2.35 (s, 3H), 1.21 (t, *J* = 7.3 Hz, 3H). ^13^C NMR (CDCl_3_) *δ*: 168.5, 159.9, 158.2, 152.6, 151.3, 145.0, 142.6, 141.0, 136.2, 129.9, 128.6, 124.0, 122.4, 121.3, 120.2, 115.7, 112.9, 55.2, 42.1, 35.3, 17.8, 13.6 ppm. HRMS (ESI-TOF) calcd. for C_22_H_22_N_4_O_2_SCl [M+H]^+^ 441.1147; found: 441.1143.

**2-Chloro-5,11-dihydro-11-ethyl-8-{[(phenyl)thio]methyl}-5-methyl-6*H*-dipyrido[3,2-*b*:2’,3’-*e*][1,4]diazepin-6-one (9).** A solution of **2** (32 mg, 0.08 mmol) in *N*,*N*-dimethylformamide (2 mL) was added with sodium hydride (60% suspension in oil, 5 mg, 0.12 mmol), and the mixture was heated at 50 °C under N_2_ atmosphere for 30 min. After cooling down, methyl iodide (0.05 mL, 0.85 mmol) was added, and the mixture was stirred at room temperature for 0.5 h. Then water was added, and the mixture was extracted with ethyl acetate. The organic layer was washed with water, dried over Na_2_SO_4_, and concentrated under reduced pressure. The residue was purified by silica gel column chromatography (20% EtOAc/hexane) to give **9** (27 mg, 81%). ^1^H NMR (CDCl_3_) *δ*: 8.24 (d, *J* = 2.2 Hz, 1H), 8.05 (d, *J* = 2.2 Hz, 1H), 7.42 (d, *J* = 8.1 Hz, 1H), 7.32–7.18 (m, 5H), 7.09 (d, *J* = 8.8 Hz, 1H), 4.15 (q, *J* = 7.3 Hz, 2H), 4.05 (s, 2H), 3.46 (s, 3H), 1.24 (t, *J* = 7.3 Hz, 3H). ^13^C NMR (CDCl_3_) *δ*: 167.0, 158.0, 154.2, 150.6, 145.0, 141.4, 135.1, 133.1(2C), 130.4(2C), 130.2, 129.0(2C), 126.9, 120.6, 119.8, 41.5, 37.4, 35.5, 13.5 ppm. HRMS (APCI-TOF) calcd. for C_21_H_20_N_4_OSCl [M+H]^+^ 411.1041; found: 411.1042.

**2-Chloro-5,11-dihydro-11-ethyl-4-methyl-8-{[(phenyl)thio]methyl}-5-methyl-6*H*-dipyrido[3,2-*b*:2’,3’-*e*][1,4]diazepin-6-one (7).** Using a procedure analogous to the preparation of **9**, and starting from **5**, **7** was obtained in 87% as white crystals. FT-IR (KBr), ν_max_: 2970, 1645, 1583, 1452, 1343, 1247, 733, 690 cm^−1^. ^1^H NMR (CDCl_3_) *δ*: 8.21 (d, *J* = 2.2 Hz, 1H), 7.99 (d, *J* = 2.2 Hz, 1H), 7.30–7.21 (m, 5H), 6.96 (s, 1H), 4.14 (q, *J* = 7.3 Hz, 1H), 4.12 (q, *J* = 7.3 Hz, 1H), 4.05 (s, 2H), 3.32 (s, 3H), 2.32 (s, 3H), 1.24 (t, *J* = 7.3 Hz, 3H). ^13^C NMR (CDCl_3_) *δ*: 168.1, 158.7, 156.8, 150.0, 146.4, 145.8, 140.8, 135.1, 130.5(2C), 129.0(2C), 128.8, 128.5, 127.0, 122.0, 121.6, 41.2, 38.2, 35.7, 19.0, 13.5 ppm. MS (EI), *m/z* (relative intensity): 425 (M^+^, 3), 349 (21), 269 (100), 227 (30). HRMS (APCI-TOF) calcd. for C_22_H_22_N_4_OSCl [M+H]^+^ 425.1197; found: 425.1192.

**2-Chloro-5,11-dihydro-11-ethyl-4-methyl-8-{[(3-methoxyphenyl)thio]­methyl}-5-methyl-6*H*-dipyrido[3,2-*b*:2’,3’-*e*][1,4]diazepin-6-one (8).** Using a procedure analogous to the preparation of **9**, and starting from **6**, **8** was obtained in 83% as white crystals; mp 120–121 °C. FT-IR (KBr), ν_max_: 2935, 1642, 1588, 1455, 1385, 1249, 782, 689 cm^−1^. ^1^H NMR (CDCl_3_) *δ*: 8.23 (d, *J* = 2.2 Hz, 1H), 8.01 (d, *J* = 2.2 Hz, 1H), 7.27–6.71 (m, 4H), 4.14 (q, *J* = 7 Hz, 2H), 4.05 (s, 2H), 3.70 (s, 3H), 3.32 (s, 3H), 2.33 (s, 3H), 1.25 (t, *J* = 7 Hz, 3H). ^13^C NMR (CDCl_3_) *δ*: 168.0, 159.9, 158.5, 156.6, 149.8, 146.5, 145.8, 141.0, 136.2, 129.9, 128.8, 128.5, 122.5, 122.1, 121.7, 115.7, 113.0, 55.2, 41.3, 38.2, 35.5, 19.0, 13.5 ppm. MS (EI), *m/z* (relative intensity): 454 (M^+^, 4), 315 (100), 287 (13). HRMS (APCI-TOF) calcd. for C_23_H_24_N_4_O_2_SCl [M+H]^+^ 455.1308; found: 455.1316.

**2-Chloro-4-methyl-3-nitropyridine (25).** A stirred mixture of **24** (600 mg, 3.90 mmol) and POCl_3_ (2 mL, 21.4 mmol) was heated at 150 °C in a sealed vessel for 6 h. After cooling down, the mixture was poured into ice-water and stirred for an additional 1 h. The resultant precipitate was filtered and washed with water. The solid was dissolved in CH_2_Cl_2_ and washed with saturated aqueous NaHCO_3_ and water, then dried over Na_2_SO_4_ and concentrated under reduced pressure. The residue was purified by silica gel column chromatography (15% EtOAc/hexane) to yield **25** (570 mg, 85%) as a yellow solid; mp 51–52 °C. FT-IR (KBr), ν_max_: 1590, 1526, 1359, 854, 838 cm^−1^. ^1^H NMR (CDCl_3_) *δ*: 8.39 (d, *J* = 5.1 Hz, 1H), 7.25 (d, *J* = 5.1 Hz, 1H), 2.40 (s, 3H). ^13^C NMR (CDCl_3_) *δ*: 149.8 (2C), 142.0, 141.8, 125.1 (2C), 17.1 ppm. MS (EI), *m/z* (relative intensity): 173 (M^+^, 100), 156 (30), 128 (6). HRMS (APCI-TOF) calcd. for C_6_H_6_N_2_O_2_Cl [M+H]^+^ 173.0112; found: 173.0113.

**2-(Cyclopropylamino)-4-methyl-3-nitropyridine (26).** A stirred mixture of **25** (550 mg, 3.2 mmol) and cyclopropylamine (0.9 mL, 12.8 mmol) in xylene (1 mL) was heated at 105 °C in a sealed vessel for 4 h. After cooling down, the mixture was poured into water and extracted with CH_2_Cl_2_. The organic layer was washed with water, dried over Na_2_SO_4_, and concentrated under reduced pressure. The residue was purified by silica gel column chromatography (10% EtOAc/hexane) to yield **26** (610 mg, 99%) as a yellow solid. FT-IR (KBr), ν_max_: 3404, 1596, 1558, 1359, 861 cm^−1^. ^1^H NMR (CDCl_3_) *δ*: 8.23 (d, *J* = 5.1 Hz, 1H), 7.51 (br s, 1H), 6.54 (d, *J* = 5.1 Hz, 1H), 3.00–2.88 (m, 1H), 2.53 (s, 3H), 0.95–0.85 (m, 1H), 0.62–0.54 (m, 1H). ^13^C NMR (CDCl_3_) *δ*: 153.6, 152.2, 146.0, 131.0, 116.3, 24.4, 21.4, 7.3 (2C) ppm. MS (EI), *m/z* (relative intensity): 194 ((M+H)^+^, 100), 176 (76), 146 (60), 131 (20).

**3-Amino-2-cyclopropylamino-4-methylpyridine (27).** Using a procedure analogous to the preparation of **14**, and starting from **26**, **27** was obtained in 83% as a white solid; mp 141–143 °C. FT-IR (KBr), ν_max_: 3334, 1655, 1609, 1558, 1516, 1424, 1365, 1235, 1019, 805 cm^−1^. ^1^H NMR (CDCl_3_) *δ*: 7.72 (d, *J* = 5.1 Hz, 1H), 6.49 (d, *J* = 5.1 Hz, 1H), 4.63 (br s, 1H), 3.19 (br s, 2H), 2.84–2.77 (m, 1H), 2.15 (s, 3H), 0.84–0.75 (m, 1H), 0.53–0.46 (m, 1H). ^13^C NMR (CDCl_3_) *δ*: 150.1, 138.0, 129.6, 127.0, 116.6, 24.5, 16.6, 7.1 (2C) ppm. MS (EI), *m/z* (relative intensity): 164 ((M+H)^+^, 100), 148 (66), 134 (20), 80 (22). HRMS (APCI-TOF) calcd. for C_9_H_14_N_3_ [M+H]^+^ 164.1182; found: 164.1182.

**2-Hydroxy-5-nitronicotinic acid (29).** A 69% HNO_3_ (3 mL, 46.3 mmol) was added to a stirred solution of **28** (3.0 g, 21.6 mmol) in concentrated H_2_SO_4_ (24 mL). The reaction mixture was heated to 50 °C and maintained at that temperature for 7 h. After cooling down, the mixture was poured into ice-water and stirred for an additional hour. The resultant pale yellow solid was filtered, washed with cold water, and dried to yield **29** (3.13 g, 79%); mp 241–242 °C. FT-IR (KBr), ν_max_: 3027, 2872, 1717, 1624, 1572, 1466, 1356, 1147, 896, 653, 599, 509 cm^−1^. ^1^H NMR (acetone-*d*_6_) *δ*: 9.16 (d, *J* = 2.9 Hz, 1H), 9.00 (d, *J* = 2.9, 1H). ^13^C NMR (acetone-*d*_6_) *δ*: 165.7, 163.7, 142.1, 140.2, 133.4, 118.3 ppm. MS (EI), *m/z* (relative intensity): 184 (M^+^, 50), 140 (100), 127 (31), 108 (24), 94 (75).

**2-Chloro-5-nitronicotinic acid (30).** A mixture of **29** (2.0 g, 10.87 mmol) and POCl_3_ (6 mL, 64.36 mmol) was heated at reflux for 4 h. After cooling down to ambient temperature, the mixture was slowly dropped into water (200 mL), maintaining the temperature below 40 °C. Ice was added as necessary to cool the solution. Afterwards, the solution was stirred at room temperature overnight. The mixture was then extracted with diethyl ether, and the organic layer was washed with water, dried over Na_2_SO_4_, and concentrated under reduced pressure to yield **30** (1.72 g, 78%) as a yellow solid; mp 140–141 °C. FT-IR (KBr), ν_max_, cm^−1^: 2909, 1718, 1609, 1575, 1415, 1358, 1273, 1240, 1135, 732. ^1^H NMR (DMSO-*d*_6_) *δ*: 9.32 (d, *J* = 2.9 Hz, 1H), 8.85 (d, *J* = 2.9, 1H). ^13^C NMR (DMSO-*d*_6_) *δ*: 163.9, 152.8, 146.5, 143.3, 134.9, 128.3 ppm. MS (EI), m/z(relative intensity): 202 (M+, 100), 174 (53), 156 (74), 100 (65), 76 (67).

**N-(2-cyclopropylamino-4-methyl-3-pyridinyl)-2-chloro-5-nitro-3-pyridinecarboxamide (31).** A stirred solution of **30** (250 mg, 1.23 mmol) in benzene (5 mL) was treated with oxalyl chloride (0.3 mL, 3.9 mmol) followed by a catalytic amount of *N*,*N*-dimethylformamide (2 drops), and the mixture was stirred at room temperature for 1 h. Then the solvent was removed under reduced pressure to produce the acid chloride as a white solid. The acid chloride was then dissolved in tetrahydrofuran (2 mL) and was added dropwise to a cooled stirred solution of **27** (116 mg, 0.71 mmol) in tetrahydrofuran (3 mL) containing *N*-ethyldiisopropylamine (0.37 mL, 2.13 mmol). The reaction mixture was stirred at room temperature for 5 h. Afterwards, the mixture was added water and extracted with CH_2_Cl_2_. The organic layer was washed with saturated aqueous NaHCO_3_ and water. It was then dried over Na_2_SO_4_ and concentrated under reduced pressure. The residue was purified by silica gel column chromatography (30% EtOAc/hexane) to produce **31** (105 mg, 53%) as a yellow solid; mp 172–173 °C (dec.). FT-IR (KBr), ν_max_: 3230, 1650, 1602, 1568, 1521, 1352, 746 cm^−1^. ^1^H NMR (acetone-*d*_6_) *δ*: 9.30 (d, *J* = 2.9 Hz, 1H), 9.22 (br s, 1H), 8.95 (d, *J* = 2.9, 1H), 7.96 (d, *J* = 5.1 Hz, 1H), 6.58 (d, *J* = 4.4 Hz, 1H), 5.80 (br s, 1H), 2.79.–2.71 (m, 1H), 2.29 (s, 3H), 0.73–0.63 (m, 2H), 0.42–0.35 (m, 2H). ^13^C NMR (acetone-*d*_6_) *δ*: 163.2, 157.1, 153.4, 147.5, 146.5, 145.6, 144.7, 134.4, 134.2, 116.9, 115.9, 25.2, 18.2, 7.5 (2C) ppm. MS (EI), *m/z* (relative intensity): 348 ((M+H)^+^, 56), 332 (15), 314 (100), 233 (65), 162 (75). HRMS (APCI-TOF) calcd. for C_15_H_15_N_5_O_3_Cl [M+H]^+^ 348.0858; found: 348.0863.

**4-Methyl-8-nitro-5,11-dihydro-11-cyclopropyl-6*H*-dipyrido[3,2-*b*:2’,3’-*e*][1,4]diazepin-6-one (32).** A suspension of **31** (378 mg, 1.09 mmol) in hexamethyldisilazane (36 mL) was heated at 110 °C for 24 h. After cooling down to room temperature, the mixture was added with water and extracted with CH_2_Cl_2_. The organic layer was washed with water, dried over Na_2_SO_4_, and concentrated under reduced pressure. Recrystallization from acetone produced yellow crystals of **32** (305 mg, 90%); mp 266–268 °C (dec.). FT-IR (KBr), ν_max_: 3197, 1659, 1592, 1519, 1460, 1336 cm^−1^. ^1^H NMR (CDCl_3_) *δ*: 9.23 (d, *J* = 2.2 Hz, 1H), 8.89 (d, *J* = 2.2 Hz, 1H), 8.20 (d, *J* = 5.1 Hz, 1H), 8.10 (br s, 1H), 7.04 (d, *J* = 5.1 Hz, 1H), 3.80–3.70 (m, 1H), 2.42 (s, 3H), 1.10–1.00 (m, 1H), 0.60–0.50 (m, 1H). ^13^C NMR (CDCl_3_) *δ*: 166.4, 163.9, 151.8, 147.5, 144.9, 140.1, 139.6, 136.5, 124.4, 123.0, 118.8, 30.8, 17.6, 9.3 (2C) ppm. HRMS (ESI-TOF) calcd. for C_15_H_14_N_5_O_3_ [M+H]^+^ 312.1091; found: 312.1087.

**8-Amino-4-methyl-5,11-dihydro-11-cyclopropyl-6H-dipyrido[3,2-*b*:2’,3’-*e*][1,4]diazepin-6-one hydrochloride salt (10).** A solution of SnCl_2_.2H_2_O (190 mg, 0.84 mmol) in concentrated HCl (0.7 mL) was added dropwise to a stirred, cooled solution of **32** (80 mg, 0.25 mmol) in acetic acid (2.8 mL). The yellow precipitate was formed, and the mixture was stirred for 1 h at room temperature. The yellow solid was collected by filtration, washed with tetrahydrofuran, and dried to produce **10** (60 mg, 73%). FT-IR (KBr), ν_max_: 3519, 2587, 1668, 1620, 1539, 1468, 1254 cm^−1^. ^1^H NMR (DMSO-*d*_6_) *δ*: 10.12 (s, 1H), 8.47 (d, *J* = 2.2 Hz, 1H), 8.12 (d, *J* = 5.1 Hz, 1H), 7.99 (d, *J* = 2.2 Hz, 1H), 7.18 (d, *J* = 5.1 Hz, 1H), 5.63 (br s), 3.63 (m, 1H), 2.37 (s, 3H), 0.92–0.89 (m, 1H), 0.50–0.30 (m, 1H). ^13^C NMR (DMSO-*d*_6_) *δ*: 165.6, 156.6, 152.6, 143.6, 142.6, 142.4, 133.4, 128.0, 125.2, 122.7, 121.1, 29.5, 17.7, 8.8, 8.6 ppm. HRMS (ESI-TOF) calcd. for C_15_H_16_N_5_O [M+H]^+^ 282.1349; found: 282.1354.

**8-Amino-4-methyl-5,11-dihydro-11-cyclopropyl-6H-dipyrido[3,2-*b*:2’,3’-*e*][1,4]diazepin-6-one (11).** A solution of **10** in water was added with 50% aqueous NaOH to pH 12 and was stirred for 1 h. Then the mixture was extracted with CH_2_Cl_2_ for several times. The combined organic layers were washed with water, dried over Na_2_SO_4_, and concentrated under reduced pressure to yield **11** (90%); mp 293–294 °C (dec.). FT-IR (KBr), ν_max_: 3362, 3317, 1662, 1640, 1602, 1472, 1237 cm^−1^. ^1^H NMR (DMSO-*d*_6_) *δ*: 9.69 (s, 1H), 8.01 (d, *J* = 4.4 Hz, 1H), 7.84 (d, *J* = 2.2 Hz, 1H), 7.20 (d, *J* = 2.9 Hz, 1H), 6.98 (d, *J* = 5.1 Hz, 1H), 5.25 (s, 2H), 3.60–3.40 (m, 1H), 2.28 (s, 3H), 0.80–0.60 (m, 1H), 0.30–0.10 (m, 1H). ^13^C NMR (DMSO-*d*_6_) *δ*: 167.4, 155.3, 149.9, 143.2, 141.6, 140.1, 137.2, 124.8, 123.1, 121.5, 121.0, 28.8, 17.5, 8.5, 8.4 ppm. HRMS (APCI-TOF) calcd. for C_15_H_16_N_5_O [M+H]^+^ 282.1360; found: 282.1352.

Biological Testing

**Reverse Transcription Reaction.** This method was adapted from Lerma et al. [1]. HIV-1_SUM9_ virus was used as a wild-type HIV-1 reverse transcriptase, and K103N and Y181C were used as the mutant HIV-1 RT enzymes. The exogenous RNA template used in the experiment was derived from the Encephalomyocarditis virus (EMCV) genome. The reaction mixture for reverse transcription is under the following conditions: 10 μL 5X RT buffer (250 mM Tris-HCl, pH 8.3, 250 mM KCl, 50 mM MgCl_2_), 5 μL of 10 mM EGTA, 1 μL of 100 mM DTT, 6 μL of 2%NP40, 2 μL of 10 μM dNTP, 4 μM EMCV R2 primer, 0.25 μL 40,000 U/mL RNasin, 0.5 μL of 20 ng/μL EMCV RNA template, and 5.3 μL of diethyl pyrocarbonate-water. Then 1 μL of wild-type or mutant type HIV-1 virus at a concentration of 10^7^ nUnits/μL or 1 μL of DMSO was added to a 19 μL reaction mixture. The mixture was then incubated at 42 °C for 1 h. In case of inhibition experiments, 1 μL of inhibitors in DMSO at different concentrations was added instead of DMSO. The reactions were stopped by heat at 95 °C for 5 min. Then the EMCV DNA products from these reactions were used as templates for real-time polymerase chain reaction (PCR). The concentrations of 10^8^–10^3^ nU/μL of AMV reverse transcriptase were used to create the EMCV cDNA product standard curve.

**Real-time Polymerase Chain Reaction (PCR).** Real-time PCR assay was detected using a Rotor-Gene 3000 (Corbett Robotics, Australia). The PCR cocktail was prepared by adding 2 μL of 10X PCR buffer, 0.5 μL of 10 μM EMCV forward and reverse primers, 0.5 μL of hot-start Taq polymerase (Qiagen), 0.25 μL of 100 μM 6-carboxylfluorescein (FAM probe), and 14.05 μL of water. Afterwards, 1 μL of the production of reverse transcription reaction was added to each tube containing 19 μL of the PCR mastermix. The conditions for PCR are held at 95 °C for 15 min, then 40 cycles of 95 °C for 20 s, 55 °C for 30 s, and 72 °C for 30 s. Fluorescence data were acquired at the end of each annealing step.

**Analysis of Percent Inhibition of Reverse Transcriptase and IC_50_.** Percent inhibition of reverse transcriptase was calculated from the difference of the reverse transcriptase activity of HIV-1 (wild-type or mutants) without inhibitor and its activity with inhibitor, divided by its activity without inhibitor, then multiplied by 100. The IC_50_ was then analyzed as described by Ferreira et al. [2]. Nonlinear regression analysis was used for fitting data to a sigmoidal concentration-response relationship Y=1/(1+10^((LogIC50-X)Slope^), where the slope is the Hill slope parameter, IC_50_ is the concentration of inhibitors that is required for 50% inhibition of reverse transcriptase, X is the compound concentration (M), and Y is 0.01 of percent inhibition (GraphPad Prism; GraphPad Software, San Diego, CA).

**References**

1. Lerma, J. G. G.; Heneine, W. Ultrasensitive detection of reverse transcriptase activity by the Amp-RT assay: Applications to the measurement of virus loads and phenotypic drug resistance. In *Antiviral Methods and Protocols;* Kinchington, D.; Schinazi, R. F., Eds.; Humana Press: Totowa, New Jersey, 2000; pp. 291–300.

2. Ferreira, S.; Crumb, W.J. Jr.; Carlton, C. G.; Clarkson, C. W. *J. Pharmacol. Exp. Ther.* **2001**, *299*(1), 220–226.
